# Benign prostatic hyperplasia surgical treatment trends in the Public Health System in São Paulo, Brazil

**DOI:** 10.31744/einstein_journal/2022AO6880

**Published:** 2022-06-14

**Authors:** Álan Roger Gomes Barbosa, Lucas Seiti Takemura, Breno Santos Amaral, Marcelo Langer Wroclawski, Wladimir Alfer, Antonio Otero Gil, José Monteiro, Danilo Budib Lourenço, Jonathan Doyun Cha, Marcelo Apezzato, João Arthur Brunhara Alves Barbosa, Bianca Bianco, Gustavo Caserta Lemos, Arie Carneiro

**Affiliations:** 1 Hospital Israelita Albert Einstein São Paulo SP Brazil Hospital Israelita Albert Einstein, São Paulo, SP, Brazil.

**Keywords:** Prostatic hyperplasia, Transurethral resection of prostate, Prostatectomy, Postoperative complications, Treatment outcome, Length of stay, Mortality

## Abstract

**Objective:**

To describe and compare the number of surgeries, mortality rate, length of hospital stay, and costs of transurethral resection of the prostate and open prostatectomy for the treatment of benign prostatic hyperplasia, between 2008 and 2018, in the Public Health System in São Paulo, Brazil.

**Methods:**

Ecological and retrospective study using data from the informatics department of the Brazilian Public Health System database. Procedure codes were “open prostatectomy” and “transurethral resection of the prostate.” The outcomes analyzed were compared between transurethral resection of the prostate and open prostatectomy according to the hospital surgical volume and presence or absence of a residency program.

**Results:**

A total of 18,874 surgeries were analyzed (77% transurethral resection of the prostate) and overall mortality was not statistically different between procedures. Intermediate and high-volume centers had shorter length of hospital stay than low-volume centers for transurethral resection of the prostate (3.28, 3.02, and 6.58 days, respectively, p=0.01 and p=0.004). Length of hospital stay was also shorter for open prostatectomy in high-volume compared to low-volume centers (4.86 *versus* 10.76 days, p=0.036). Intrahospital mortality was inversely associated with surgical volume for transurethral resection of the prostate. Centers with residency program had shorter length of hospital stay considering open prostatectomy and less mortality regarding transurethral resection of the prostate. Open prostatectomy was 64% more expensive than transurethral resection of the prostate.

**Conclusion:**

The findings suggest the importance of investing in specialized centers, which could be potential referral centers for surgical cases.

## INTRODUCTION

Benign prostatic hyperplasia (BPH) is defined by the increased number of cells in the epithelium and prostatic stroma, mainly localized in the transition zone of the prostate.^(1)^ The prevalence of BPH increases progressively with age, estimated as 8% in men aged 31-40 years, 40%-50% in 51-60 years, and up to 80% in those >80 years.^(2)^

Benign prostatic hyperplasia is the most common cause of bladder outlet obstruction, which leads to lower urinary tract symptoms (LUTS), such as increased urinary frequency, acute urinary retention, and urinary urgency, which negatively affects quality of life. Furthermore, voiding issues can cause serious complications in more advanced stages, such as refractory bleeding, recurrent urinary infection, post renal failure, and retrograde ejaculation.^(2-5)^

Approximately 30% of individuals with LUTS due to BPH require clinical treatment, with 20% requiring a surgical procedure, generally reserved for clinical refractory cases and the abovementioned complications.^(3,6)^ Although symptomatology is not proportional to the prostate size,^(7)^ the prostate volume is often a determining factor for choosing the surgical approach to treat BPH.^(8)^

There are several surgical modalities for BPH, which are traditionally divided into simple prostatectomy, indicated for prostates above 80g, and endoscopic procedures, usually indicated for prostates up to 80g. Among endoscopic surgeries, the most used technique is transurethral resection of the prostate (TURP), either with a mono or bipolar electrocautery.^(9,10)^Regarding simple prostatectomy, the most frequently performed procedure is the open surgery, in a suprapubic (Freyer) or retropubic (Millin) fashion.^(11)^ Nowadays, it can also be performed through a minimally invasive method, either purely laparoscopic or robotic assisted.

Surgical treatment of BPH is usually highly effective; however, all surgical acts may have immediate or late complications inherent to the procedure. These include bleeding, requirement for blood transfusion, prolonged length of hospital stay (LOHS), urinary retention, delayed bladder catheterization, urinary tract infection, and surgical site infection. These events impact the costs of BPH surgical treatment.

Brazil has a population of about 210 million people, with approximately 70% assisted by the Public Health System (SUS - *Sistema* Único *de Saúde*),^(12)^ a government organization funded by taxes. São Paulo is the largest city in Brazil and the eighth most populated city in the world. It is estimated that in 2016, there were more than 12 million inhabitants, with more than 5 million exclusively dependent on SUS. Hospitals of SUS bill the procedures according to codes and receive a predetermined fixed amount of reimbursement for each code. Due to restricted resources, SUS patients mainly undergo only two surgical approaches to BPH: TURP and open prostatectomy (OP).

Understanding the trends of BPH-related procedures on a state and regional level is important in evaluating how we can improve delivery of efficient, cost-effective, and high-quality health care, and also can help identify dynamic associations and *deficits* in patient care.^(13)^

## OBJECTIVE

To describe and compare the number of surgeries, intrahospital mortality rates, length of hospital stay, and costs of transurethral resection of the prostate and open prostatectomy, between 2008 and 2018, in the Public Health System in São Paulo, Brazil, and to compare data between low, intermediate, and high-volume centers, and the presence or absence of a Medical Residency Program in Urology.

## METHODS

This was an ecological retrospective study, conducted using the TabNet Platform and Brazilian Public Health System database (DATASUS - *Departamento de Informática do Sistema Único de Saúde do Brasil*), between 2008 and 2018. This database consists of open data about procedures performed in the Brazilian SUS, which can be found online at http://www2.datasus.gov.br. Procedure codes used for this study were “OP” (code 04.09.03.002-3) and “TURP” (code 04.09.03.004-0). We chose a 10-year span to detect consistent trends, as well as to overcome limitations and biases due to transitory variations that could possibly occur in the public healthcare system.

Outcomes analyzed included number of surgeries, mortality rate during hospital stay, LOHS, length of intensive care unit (ICU) stay, and costs. Cost was calculated as the total amount paid annually to each institution for each procedure, divided by the total number of hospitalizations related to the same procedure. The specific amount paid by the public health for each procedure is not available for analysis. These results were compared between TURP and OP, and according to the hospital surgical volumes. For this analysis, centers were divided into thirds. Additionally, the presence or absence of a Medical Residency Program in Urology was evaluated.

### Statistical analysis

After data extraction, the results were organized and pooled in Microsoft Office Excel 2016^®^ (v. 16.0.4456.1003, Redmond, Washington, USA). Data normality was verified using Kolmogorov-Smirnov test. Mann-Whitney test was used to compare non-normal variables (ICU hospitalization days), and *t*-test for variables with normal distribution (LOHS and total costs). Statistical significance was considered when p<0.05.

The Research Ethics Committee from the *Hospital Israelita Albert Einstein* (HIAE) approved this study, with reference # 3.625.161, CAAE: 17208019.0.0000.0071.

## RESULTS

### Trends in type of surgery over time

**Figure 1:**
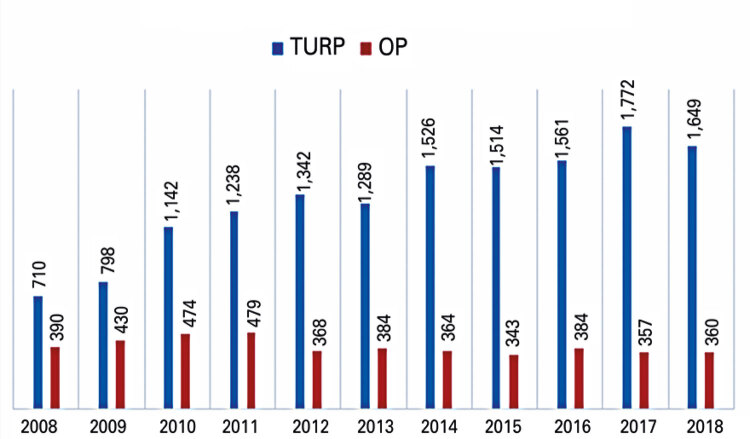
Number of procedures annually

OP: open prostatectomy; TURP: transurethral resection of the prostate.

A total of 18,874 surgeries were analyzed between 2008 and 2018, with 14,511 TURPs (77%) and 4,333 OPs (23%). Considering TURP, 38 institutions performed these procedures, of which 15 (39.4%) had Medical Residency Program in Urology. Regarding OPs, 40 institutions were included, of which 15 (37.5%) had Medical Residency Program in Urology. The percentage of endoscopic surgeries has progressively increased over the years. In 2008, 1,100 surgeries due to BPH were performed, and 65% of them were TURPs. In 2018, a total of 2,009 surgeries were performed, with 82% of TURP, which is a statistically significant increase (p<001). Figure 1 shows the number of procedures annually, from 2008 to 2018.

### Description and classification of the institutions analyzed

According to the number of surgeries, institutions were divided into low, intermediate, and high-volume centers. The number of institutions in the low, intermediate, and high-volume center categories for TURP was, 13, 12, and 13, respectively; whereas for OP it was, 13, 13, and 14, respectively. Considering TURP, low volume centers had 1 to 76 surgeries in the period (median of 9 cases/institution/per 10 years); intermediate centers had 83 to 257 surgeries (median of 182.5 cases/institution/per 10 years) and high volume centers had 261 to 2,754 (median of 740 cases/institution/per 10 years). As for OP, low, intermediate and high-volume centers had 1 to 15 procedures (median of 7 cases/institution/per 10 years), 25 to 63 procedures (median of 31 cases/institution/per 10 years), and 83 to 734 surgeries (median of 185.5 cases/institution/per 10 years), respectively in the same period.

### Analysis of length of stay

Length of hospital stay was shorter for patients who underwent TURP than in those who underwent OP (3.04 *versus* 5.19 days; p<0.01). For TURP, LOHS was significantly shorter for high-volume centers compared to low-volume centers (3.02 *versus* 6.58 days, p=0.004) and for intermediate-volume centers compared to low-volume centers (3.28 *versus* 6.58 days, p=0.01). Regarding OP, LOHS was shorter in high-volume centers compared to low-volume centers (4.86 *versus* 10.76 days, p=0.036) (Table 1) and also in institutions with Medical Residency Program in Urology (4.98 *versus* 8.63; p=0.004) (Table 2).

**Table 1:** Length of hospital stay, intensive care unit stay, mortality, and costs of transurethral resection of the prostate and open prostatectomy analyzed among low, intermediate, and high-volume centers

Variables	TURP				OP			
	Low volume	Intermediate volume	High volume	p value^†^	Low volume	Intermediate volume	High volume	p value^†^
Days of hospital stay*	6.58±4.38	3.28±0.9	3.02±0.68	0.002^‡^	10.76±9.77	6.43±1.95	4.86±1.13	0.03^£^
Days of ICU stay*	0.75±1.87	0.10±0.10	0.10±0.07	0.24	0.49±0.78	0.29±0.20	0.14±0.13	0.18
Intrahospital mortality, n (%)^#^	2/330 (0.60)	9/2,093 (0.43)	19/12,118 (0.16)	0.01^&^	0/86 (0)	1/483 (0.21)	14/3,764 (0.37)	0.40
Costs*	US$ 302±297	US$ 189±31	US$ 179±19	0.15	US$ 314±63	US$ 311±63	US$ 296±38	0.34

* These variables are presented as mean and standard deviation; ^#^ This variable is presented as number/total of surgeries and percentage (%); ^†^ ANOVA test with post-hoc Tukey test; ^‡^ Regarding transurethral resection of the prostate, days of hospital stay showed significant difference between low and medium volume (p=0.01) and low and high-volume (p=0.004) centers. ^£^ Considering open prostatectomy, days of hospital stay showed significant difference between low and high-volume (p=0.036). ^&^ Regarding TURP, intrahospital mortality showed significant difference between intermediate and high-volume (p=0.019).

Results expressed as mean±standard deviation or n (%).

OP: open prostatectomy; TURP: transurethral resection of the prostate; ICU: intensive care unit.

**Table 2:** Length of hospital stay, intensive care unit stay, mortality, and costs of transurethral resection of the prostate and open prostatectomy analyzed among institutions with or without a residency program in urology

Variables	TURP			OP		
	With Medical Residency Program in Urology	Without Medical Residency Program in Urology	p value	With Medical Residency Program in Urology	Without Medical Residency Program in Urology	p value
Days of hospital stay*	4.33±3.37	4.31±2.91	0.55	4.98±2	8.63±7.3	0.004
Days of ICU stay*	0.24±0.67	0.37±1.33	0.91	0.23±0.38	0.34±0.54	0.260
Intrahospital mortality, n (%)^#^	17/10,110 (0.17)	16/4,431 (0.36)	0.02	12/3,304 (0.36)	3/1,036 (0.29)	0.72
Costs*	US$ 213±112	US$ 223±202	0.41	US$ 294±52	US$ 301±57	0.555

* These variables are presented as mean and standard deviation; ^#^ This variable is presented as number/total of surgeries and percentage (%).

Results expressed as mean±standard deviation or n (%).

OP: open prostatectomy; TURP: transurethral resection of the prostate; ICU: intensive care unit.

Both TURP and OP had low length of ICU stay in the three groups (TURP: 0.75, 0.10, and 0.10 days and OP: 0.49, 0.29, and 0.14 days, for low, intermediate, and high-volume centers, respectively).

### Mortality analysis

Mortality rate was low in both procedures (0.24%), and more prevalent for open surgery, although this difference was not statistically significant (0.35% *versus* 0.21%; p=0.1). Intrahospital mortality was significantly different for TURP across different volume institutions (0.60% for low-volume; 0.43% for intermediate-volume and 0.16% for high-volume, p=0.01) but not significant for OP. Intrahospital mortality for TURP was also lower in centers with a Medical Residency Program in Urology (0.17% *versus* 0.36%; p=0.02) (Table 2).

### Cost analysis

Open prostatectomy is 64% more expensive than TURP, with a mean cost of US$ 298,60 per surgery, whereas each TURP costs US$ 182,10. Although both procedures cost less in centers with higher surgical volume, the results were not statistically significant (Table 1).

## DISCUSSION

This study reports the trends of BPH surgery in the Public Healthcare System of the largest Brazilian city in a period of 10 years. In our data, the use of minimally invasive procedures, namely with TURP, consistently increased in the period studied. Furthermore, high-volume centers significantly outperformed smaller ones regarding length of stay and mortality. These findings highlight the importance of organizing referral centers for concentrating procedures in high-performance facilities.

Open prostatectomy and TURP are relatively common procedures for the treatment of BPH. From 2008 to 2018, there was a consistent increase in the number of TURP procedure in SUS in São Paulo, Brazil. This may reflect progressive access to medical technology among public institutions in low-to-middle-income countries. Another possible explanation is that because of improvements in the cross-referencing system that occurred in this period, patients get to be treated earlier, before their prostate size requires an OP.

Regarding the safety outcomes of the techniques, both OP and TURP are considered safe, with low intrahospital mortality rate. This rate may be even lower in patients who undergo TURP, although this difference was not statistically significant. Open prostatectomy presents a significant morbidity rate, which justifies the longer LOHS and length of ICU stay. Certainly, indications and patient conditions are different between TURP and OP. An important aspect of this study is to demonstrate the impact of surgical volume on better outcomes. When comparing low, intermediate, and high-volume centers, those with higher surgical volume presented lower intrahospital mortality rates and shorter LOHS. Conversely, the cost per procedure in higher surgical centers was lower, but with no statistical significance.

These findings are consistent with the literature evidence. Although OP is related to increased morbidity and mortality^(14)^ studies showed low numbers.^(15)^ This study revealed the LOHS as 5 to 10 days, with reduced duration at higher volume centers. Perioperative mortality for OP was described as almost 0% in a review published in 2015.^(16)^ For TURP, as 0.4% in an American review analysis, among 4,794 surgeries included.^(17)^ Ferretti et al.^(16)^ reported median LOHS for OP was 7.4 days,^(16)^ and Zargooshi^(18)^ reported median LOHS of 7 days in 3,000 consecutives OP.

The evidence of higher volume centers with better outcomes is extremely important, considering limited resources in SUS. This evidence was predicted by our group since centers with a higher number of surgeries tend to have more resources, more technology, specialized staff, partnership with academic centers and universities, and probably, an enhanced surgical technique because of repetitive procedures. The observation that high-volume centers presented shorter LOHS and reduced mortality for a given type of surgery has fundamental implications to the organization of the public healthcare system. The advantage of reduced mortality is obvious, but also in terms of reduced LOHS, a shorter hospital stay may imply lower costs as well as presumably lesser morbidity from prolonged hospitalization complications (venous thromboembolism, infection, among others). Due to the nature of this study, we were not able to assess the occurrence of those complications, or to evaluate actual costs associated with treatment, but only to estimate these figures. This is a possible reason why we were not able to detect a statistical difference in costs of these procedures. Nevertheless, the evidence presented in this study strongly advises healthcare systems administrators to concentrate these procedures in referral centers to reduce costs and improve outcomes.

Cost of BPH treatment is of paramount importance for the SUS. In the USA, treatment costs for BPH have been estimated in approximately US$ 4 billion annually,^(19)^ and it is expected that this cost tends to increase in the future, due to aging of the population.^(20)^

Hospitals with Medical Residency Program in Urology had shorter LOHS considering OP and less mortality considering TURP. This result is of utmost importance because despite surgeons being in the beginning of their learning curve, hospitals with an academic purpose tend to have more specialized staff members, who can guide the residents’ practice.

This study has some limitations, such as data source, which was restricted to the SUS database, depending on the notification of the procedures and outcomes by each institution. In the database system, we had access to limited outcomes. Mortality data in the database was related only to the period of hospitalization due to the procedure, and that occurring after discharge was not considered for this analysis. Another considerable point is the inner condition of each procedure: OP tends to be associated with higher morbidity because of its classic indication for larger prostates. Therefore, we assume that OP evolves with increased bleeding, LOHS, and possible mortality. Finally, for analysis of costs, access to the total amount of government funding allocated to each hospital is limited. For this analysis, costs were defined as the total amount of money transferred to each hospital for the procedure under investigation, divided by the total number of surgeries in the same period.

## CONCLUSION

The number of benign prostatic hyperplasia surgeries increased annually between 2008 and 2018. Transurethral resection of the prostate was gradually more frequently performed than open prostatectomy. Intrahospital mortality was low in both procedures. Centers with a higher volume of surgery had better outcomes in terms of length of hospital stay and mortality. Centers with Medical Residency Program in Urology had shorter length of hospital stay considering open prostatectomy, and less mortality considering transurethral resection of the prostate. These findings are in accordance with those from the developed countries and suggest the importance of investing in specialized centers, which could be potential referral centers for surgical cases.
